# RAD18 may function as a predictor of response to preoperative concurrent chemoradiotherapy in patients with locally advanced rectal cancer through caspase‐9‐caspase‐3‐dependent apoptotic pathway

**DOI:** 10.1002/cam4.2203

**Published:** 2019-04-29

**Authors:** Xueqi Yan, Jie Chen, You Meng, Chao He, Shitao Zou, Peng Li, Ming Chen, Jinchang Wu, Wei‐Qun Ding, Jundong Zhou

**Affiliations:** ^1^ Suzhou Cancer Center Core Laboratory Nanjing Medical University Affiliated Suzhou Hospital Suzhou Jiangsu PR China; ^2^ Department of Oncology The Jiangyin Clinical College of Xuzhou Medical University Wuxi Jiangsu P.R. China; ^3^ Department of Surgical Oncology Nanjing Medical University Affiliated Suzhou Hospital Suzhou Jiangsu PR China; ^4^ Department of Pathology University of Oklahoma Health Science Center Oklahoma City Oklahoma USA

**Keywords:** apoptosis, locally advanced rectal cancer, neoadjuvant chemoradiotherapy, RAD18

## Abstract

Neoadjuvant chemoradiotherapy (nCRT) has been widely applied to improve the local control rate and survival rate in patients with locally advanced rectal cancer (LARC), yet only part of LARC patients would benefit from nCRT. Therefore, it is imperative to predict the therapeutic outcome of nCRT. Here, we showed that RAD18, an E3 ubiquitin‐linked enzyme, played a fundamental role in predicting the response of LARC patients to nCRT. According to clinical data, patients with low RAD18 expression level in their pre‐nCRT biopsies had a superior response to nCRT compared to those with high RAD18 expression. Inhibition of RAD18 expression in rectal cancer cells pronouncedly attenuated the proliferation and promoted apoptosis after exposing to irradiation or/and 5‐fluorouracil (5‐Fu). Downregulated RAD18 levels increased cell apoptosis by activating caspase‐9‐caspase‐3‐mediated apoptotic pathway, thus resulting in the enhancement of cell radiosensitivity and 5‐Fu susceptibility. Furthermore, a xenograft nude mouse model showed that silencing RAD18 significantly slowed tumor growth after irradiation or/and 5‐Fu in vivo. Collectively, these results implied that RAD18 could be a new biomarker to predict LARC patients who might benefit from nCRT and provide new strategies for clinical treatment of LARC.

## 1 INTRODUCTION

Rectal cancer is one of the most common human malignant cancers in the world and the morbidity is increasing at 4.2% per year in China.^1^ Currently, the standardized treatment for locally advanced rectal cancer (LARC) is 5‐fluorouracil (5‐Fu)‐based neoadjuvant chemoradiotherapy (nCRT) followed by total mesorectal excision, which decreases the local recurrence rate and increases the complete pathologic response and tumor resectability.^2^, ^3^ Unfortunately, only 4%‐20% of these patients can achieve pathologically complete response to nCRT.^4^, ^5^ Unresponsive patients will not benefit from nCRT, and may experience severe side effects attributing to delayed surgical intervention.^6^, ^7^ Hence, it is necessary to predict the response of nCRT to distinguish the LARC patients who might benefit from nCRT. Many potential molecular biomarkers have been evaluated, yet the study findings were still controversial.^8^ More effective molecular markers are still needed to predict nCRT response.

RAD18, an E3 ubiquitin‐linked enzyme, contributes to the maintenance of genome stability and cell survival through multiple DNA repair pathways, including translesion synthesis repair pathway^9^, ^10^ and homologous recombination repair,^11^ etc. In human cancers, RAD18 has been described as a double‐edged sword since it efficiently repairs the mutagenic DNA damage in response to various genomic insults, including chemoradiotherapy, thus resulting in chemoradio‐resistance.^12^ Previous studies have shown that high expression of RAD18 confers resistance to chemotherapy or radiotherapy in multiple human cancers.^11^, ^13^, ^14^, ^15^, ^16^ However, whether expression level of RAD18 affects the response of nCRT in LARC remains obscure.

Here, we showed that low expression level of RAD18 in biopsy specimens of LARC patients correlated with good response to nCRT. RAD18 downregulation sensitized rectal cancer cells to nCRT both in vitro and in vivo. Furthermore, downregulated RAD18 promoted chemoradiosensitivity in LARC by activating caspase‐9 and caspase‐3, proteins that are important in chemoradiation‐induced cell apoptotic pathway. In conclusion, these results gave evidence that RAD18 could be a new biomarker to predict the response of nCRT in LARC and provide new strategies to improve the therapeutic effect of standard treatment in LARC patients.

## 2 MATERIALS AND METHODS

### 2.1 Patient tissues

Tissue samples of 83 LARC patients showed in the study were acquired from the Nanjing Medical University Affiliated Suzhou Hospital from 2010 to 2017 (Suzhou, Jiangsu, China). The 83 LARC patients were all received nCRT followed by operation. Among the 83 patients, only 51 patients had both pretreatment biopsy specimen and postoperative specimen used for immunohistochemistry analysis. The tissue samples were embedded in paraffin. The study was approved by the Institutional Ethics Committee of the Nanjing Medical University.

### 2.2 Cell culture and irradiation

The human colorectal cancer cell lines, HCT‐116 and DLD‐1, were acquired from the Shanghai Cell Bank (Shanghai, China). HCT‐116 and DLD‐1 cells were separately cultured in DMEM medium and RPMI‐1640 medium (Hyclone, Logan, UT, USA). All media were added with 10% FBS (Hyclone). Cells were cultured in humidified atmosphere, with 5% CO_2_ at 37°C. A 6 MV X‐ray linear accelerator at 100 cm source‐skin distance at room temperature was performed for cells in radiation groups.

### 2.3 Immunohistochemical analyses

Tumor tissue sections were deparaffinized and heat‐treated by citrate buffer, pH 6.0, for 5 minutes and treated with 0.03% hydrogen peroxide for 5 minutes. Then after incubated with diluted RAD18 antibody (1:100, (Abcam, Cambridge, MA, USA)) for 2 hours and horseradish peroxidase (HRP)‐conjugated anti‐mouse/rabbit antibody for another 1 hour at 37°C, the color was developed by 3‐3′‐diaminobenzidine. Hematoxylin was used for counterstaining. Then sections were washed and dunked momently in water containing ammonia, prior to dehydration and mounting in Diatex. The stained sections were observed by a Leica microscope (Leica, Wetzlar, Germany).

The expression level of RAD18 was scored by two pathologists. The dyeing intensity was scored as “0” (no dyeing), “1” (negative or weakly dyeing), “2” (buffy dyeing), and “3” (strong brown dyeing). The average dyeing cells rates was scored as: 0 (<10%), 1 (11%‐25%), 2 (26%‐50%), 3 (50%‐75%), and 4 (>75%). The scores of two parameters above were multiplied as the final score for each section. The expression level of RAD18 was established as high expression (score ≥6) and low expression (score <6).

### 2.4 The human colorectal carcinoma cell transfection

The shRNA targeting RAD18 (shRNA RAD18) and nontargeting control shRNA (shRNA NC) (Guangzhou RIBOBIO, Guangzhou, China) were inserted into the lentivirus expression vectors (GenePharma, Shanghai, China) and packed to viral particles which were used to infect HCT‐116 and DLD‐1 cells. Cells were collected after 3 days of transfection, then screened for 7 days with a medium containing 1 μg/mL puromycin (Sigma‐Aldrich, St. Louis, USA). Western blot was used to detect the transfection efficiency.

### 2.5 CCK‐8 proliferation assay

Cells were cultivated in 96‐well culture plates at 2000/100 μL per well and attached for 24 hours followed by treating with 5‐Fu of different concentrations (0, 5, 10, 20, 40 μg/mL) for 48 hours. Then the cells were cultured in fresh new medium with 10% CCK‐8 (Beyotime Biotechnology, Shanghai, China) each well at 37°C for 2 hours. Absorbance was detected at 450 nm by a microplate reader (Thermo, USA), and then we counted IC50 (concentration of 5‐Fu with 50% cell inhibition) in each group.

### 2.6 Colony formation assay

Cells were cultivated in 6‐well culture plates at 200, 1000, 2000 cells, respectively, per well for exposing to 0, 2, 4 Gy of radiation. After being attached for 24 hours, cells were treated with or without 5‐Fu for 24 hours followed by exposure to gradient doses (0, 2, 4 Gy) of radiation. After cultivation for 10‐14 d, colonies were dyed with crystal violet reagent (Beyotime Biotechnology). The plates were pictured using a digital camera, and the surviving colonies (colonies containing more than 50 cells under a microscope in ×100 magnification) were counted by Adobe Photoshop CC2018 (Adobe, San Jose, USA). Cell survival histogram was fitted by GraphPad Prism 7 (GraphPad Software, Inc La Jolla, USA).

### 2.7 Fluorescence intensity of γH2AX

Cells fixed by 4% paraformaldehyde were permeabilized by 0.2% Triton‐100 and blocked with 10% normal goat serum. Then cells were incubated with anti‐γH2AX antibody (Abcam, Cambridge, MA, USA) for 20 minutes at room temperature followed by incubation with the secondary antibodies for 1 hour. The fluorescence of γH2AX was detected by the LEICA DMi8 and LEICA TCS SP8. The γH2AX level was ensured by comparing the mean γH2AX fluorescence intensity in different treatment group.

### 2.8 Annexin V/7‐AAD flow cytometry assay

Cells were collected at 24 hours after 4 Gy of x‐ray radiation and washed by PBS twice and then resuspended in binding buffer and dyeing with annexin and 7‐AAD by PE Annexin V Apoptosis Detection Kit (Baosai Biotech, Shanghai, China) in dark for 15 minutes at room temperature. Apoptotic cells were detected on a FACS flow cytometer (Miltenyi, Germany) immediately.

### 2.9 Western blotting analysis

Cells were collected and lysed in RIPA buffer at 4°C (Beyotime Biotechnology) for 20 minutes. The protein concentration was detected by BCA assay (Pierce, Rockford, USA). Proteins were separated by SurePAGE^™^ precast polyacrylamide gels with a gradient between 4% and 20% (GenScript, Nanjing, China) and transferred to polyvinylidene fluoride membranes (Millipore, Billerica, MA, USA). After blocking with 5% nonfat milk, the membranes were incubated with primary antibodies against RAD18, caspase‐9, cleaved‐caspase‐9, caspase‐3, cleaved‐caspase‐3 (Abcam, USA), and β‐actin (Beyotime Biotechnology) at 4°C overnight. Then HRP‐conjugated anti‐rabbit/mouse secondary antibody (Beyotime Biotechnology) was used to incubate membranes for 1 hour. The bands were detected by enhanced chemiluminescence (Beyotime Biotechnology). Endogenous β‐actin was detected as loading control.

### 2.10 Murine xenograft models

Four‐week‐old female nude mice were acquired from Shanghai SLAC Laboratory Animal Co. Ltd. (Shanghai, China). 1 × 10^6^ cells suspended in 100 μL PBS were transferred into nude mice at left flank region by subcutaneous injection. Once the tumor grew up to 200 mm^3^ on average, the mice were divided into four groups (six mice per group). The mice were intraperitoneally injected with 10 mg/kg 5‐Fu or PBS each day for 3 days and followed or not by 10 Gy doses of irradiation at tumors.^17^ Tumor size was measured by caliper every 2 days. The volume of tumor = 4/3 × 3.14 × [(long diameter/2) (short diameter/2)^2^]. All of the mice were executed after 2 weeks. Tumors were evaluated by hematoxylin and eosin (HE) staining and examined under microscope. The animal experiments were approved by the Ethics Committee of the Nanjing Medical University.

### 2.11 Statistical analysis

Student's t test and chi‐squared test were performed using GraphPad Prism 6 software and SPSS 19 software (IBM, USA). The experiments were independently repeated for three times. The data were shown as the format of mean ± standard deviation. Statistical significance was considered at a *P*‐value < 0.05.

## 3 RESULTS

### 3.1 Low expression of RAD18 in pretreatment biopsy specimens attested clinical response to nCRT in LARC patients

**Figure 1:**
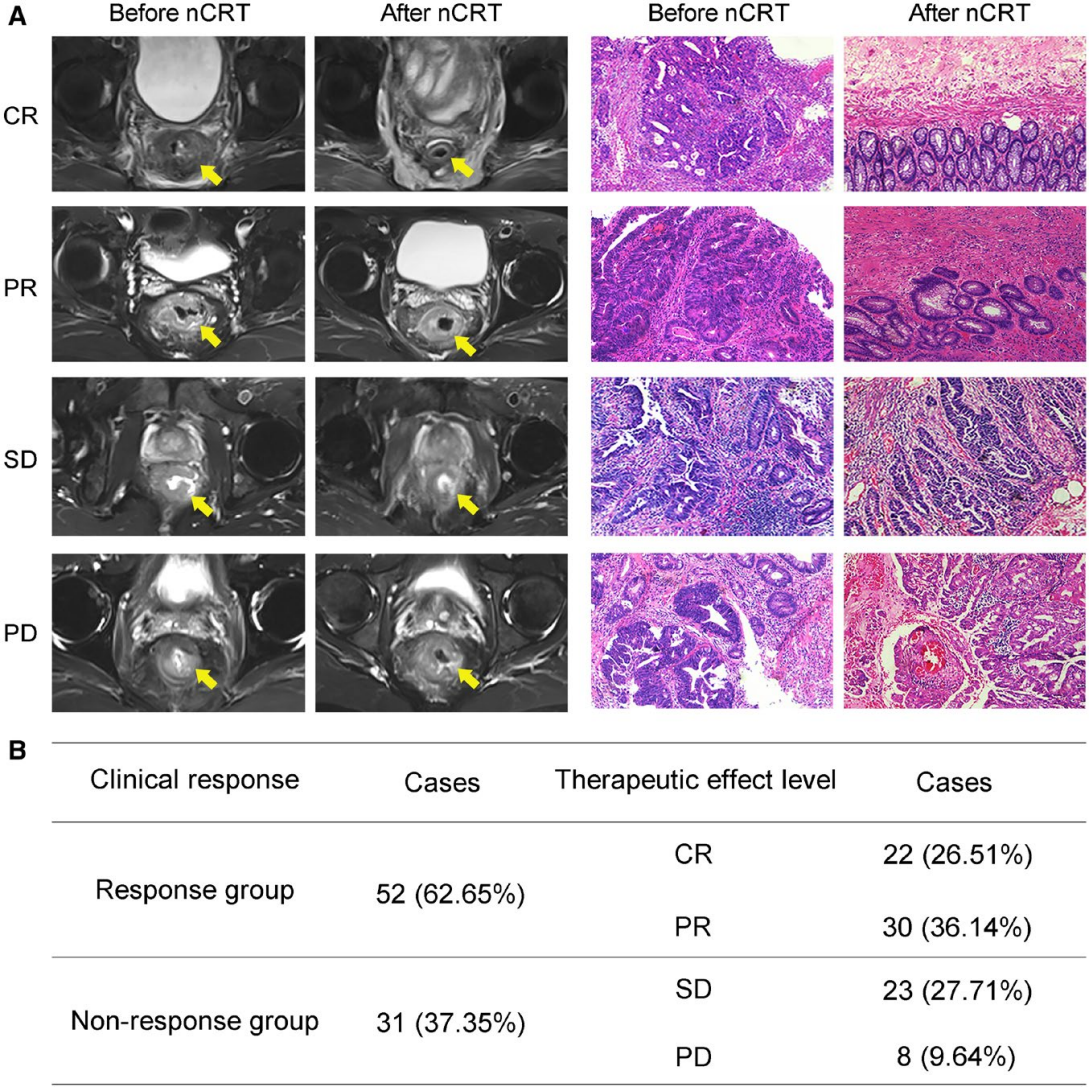
Multiple responses to nCRT were diagnosed by MRI and pathological section. A, According to the RECIST1.1 standard [Complete Response (CR): Disappearance of all target lesions and any pathological lymph nodes must have reduction in short axis to <10 mm. Partial Response (PR): At least a 30% decrease in the sum of diameters of target lesions. Stable Disease (SD): Neither sufficient shrinkage to qualify for PR nor sufficient increase to qualify for PD. Progression Disease (PD): At least a 20% increase in the sum of diameters of target lesions] and TRG for reference. Eighty‐three patients with LARC who have received nCRT treatment were divided into four groups: CR, PR, SD, PD. Representative pre‐ and post‐nCRT pelvic MRI and pathological sections (×40 magnification) of four patients who have achieved CR, PR, SD, and PD were shown. B, The percentages of CR, PR, SD, PD were 26.51%, 36.14%, 27.71%, 9.64%, respectively. The CR and PR groups were classified into response group. The SD and PD groups were classified into nonresponse group. LARC, locally advanced rectal cancer; MRI, magnetic resonance imaging; Ncrt, neoadjuvant chemoradiotherapy; TRG, tumor regression grading

To explore the relationship between RAD18 and clinical response to nCRT, 83 patients with LARC who have received nCRT were enrolled in this study. As shown in Figure 1A, according to the RECIST guideline (version 1.1) and pathological tumor regression grading (TRG, Dworak Classification),^18^ by comparing the tumor size changed on magnetic resonance imaging (MRI) and HE staining of pathological sections before and after nCRT, patients were typically separated into four groups: complete remission (CR, 22, 26.51%), partial remission (PR, 30, 36.14%), stable disease (SD, 23, 27.71%), and progressive disease (PD, 8, 9.64%). We further combined CR and PR to one category as nCRT response group (52/83, 62.65%), SD and PD to another category as nCRT nonresponse group (31/83, 37.35%) (Figure 1B). The clinical data of 83 patients indicated that the primary tumor size and lymph node metastasis were related to the response to nCRT (*P* < 0.05, Table 1).

**Table 1:** Correlations between the patient clinical characteristics and clinical response to nCRT in 83 cases of LARC

Characteristics	Cases	Clinical response		*P*‐Value
		Response group (n = 52)	Non‐response group (n = 31)	
Gender				0.143
Male	57	39 (68.4)	18 (31.6)	
Female	26	13 (50.0)	13(50.0)	
Age (year)				0.82
≤60	37	24 (64.9)	13 (35.1)	
>60	46	28 (60.9)	18 (39.1)	
Tumor size (cm)				0.022*
≤3	44	33 (75.0)	11 (25.0)	
>3	39	19 (48.7)	20 (51.3)	
Distance between lower border of tumor and anal verge (cm)				0.821
≤5	45	29 (64.4)	16 (35.6)	
>5	38	23 (60.5)	15 (39.5)	
Lymph node metastasis				0.035*
Yes	51	27 (52.9)	24 (47.1)	
No	32	25 (78.1)	7 (21.9)	
TMN stage				0.494
II	34	23 (67.6)	11 (32.4)	
III‐IV	49	29 (59.2)	20 (40.8)	
CEA (ng/mL)				
≤5	40	23 (57.5)	17 (42.5)	0.373
>5	43	29 (67.4)	14 (32.6)	
CA‐199 (U/mL)				
≤27	51	35 (68.6)	16 (31.4)	0.17
>27	32	17 (53.1)	15 (46.9)	

Abbreviations: LARC, locally advanced rectal cancer; nCRT, neoadjuvant chemoradiotherapy; TNM, tumor‐metastasis‐nodes.

**Figure 2:**
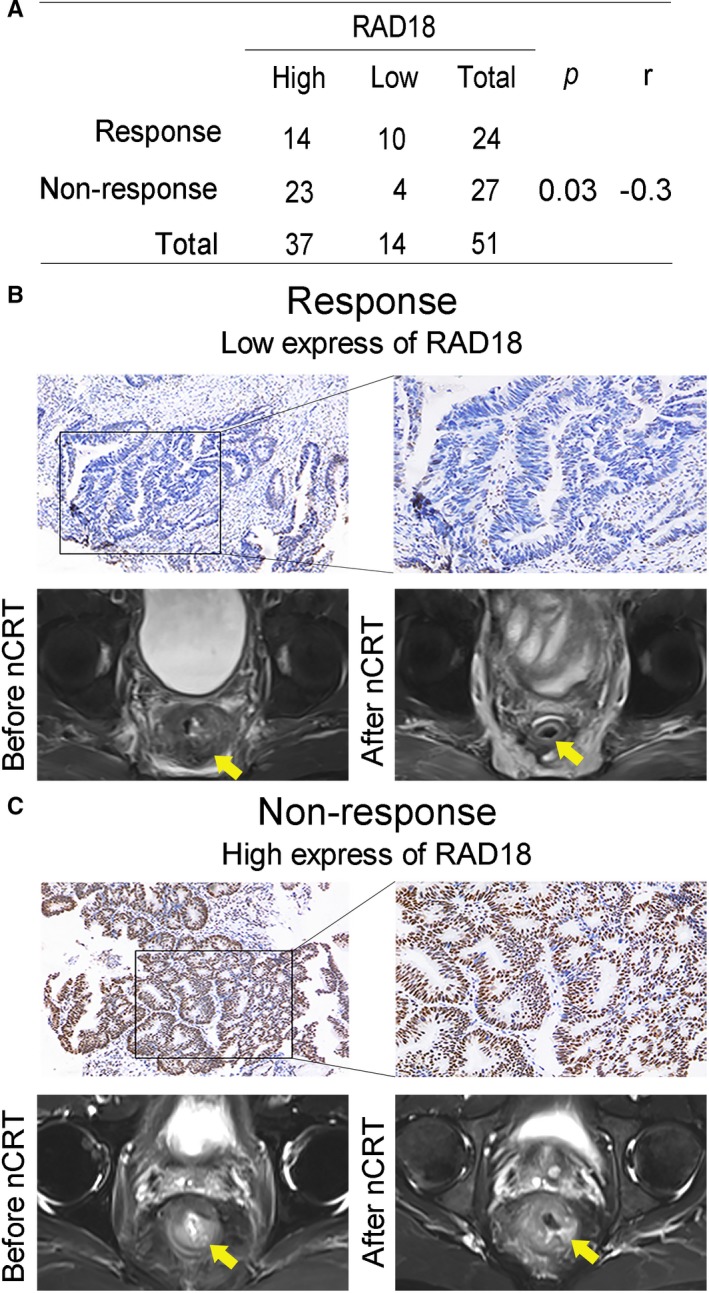
Expression of RAD18 in pretreatment biopsy specimens indicated different clinical responses to nCRT in locally advanced rectal cancer patients. A, RAD18 expression level was negatively correlated with the curative effect of nCRT in LARC patients as evidenced by chi‐squared test (*P* = 0.03). B and C, Immunohistochemical staining of RAD18 in tissue samples of patients with LARC (×40, ×200 magnification). Representative low and high expression of RAD18 and the corresponding pelvic MRI of patients were displayed. B, Low expression of RAD18; C, High expression of RAD18. LARC, locally advanced rectal cancer; Ncrt, neoadjuvant chemoradiotherapy

Among the 83 LARC patients, only 51 patients had pretreatment biopsy specimen which can be carried on immunohistochemistry analysis to detect the expression of RAD18. The nCRT sensitivity group (24/51, 47.06%) and nCRT resistance group (27/51, 52.94%) were observed. The positive staining of RAD18 was primarily located at the nucleus of rectal cancer cell. High RAD18 expression was displayed in 37 of the 51 cases (72.5%), while low RAD18 expression was shown in 14 cases (27.5%). The patients with low expression of RAD18 showed evidently tumor regression on MRI compared to those with high expression, thus indicating that RAD18 expression level was negatively correlated with the nCRT effect (*P* < 0.05, Figure 2A). Representative low and high expression of RAD18 in tissue samples by immunohistochemical (IHC) staining and the corresponding pelvic MRI are shown in Figure 2B,C.

### 3.2 Inhibition of RAD18 increased chemoradiosensitivity of rectal cancer cells in vitro

**Figure 3:**
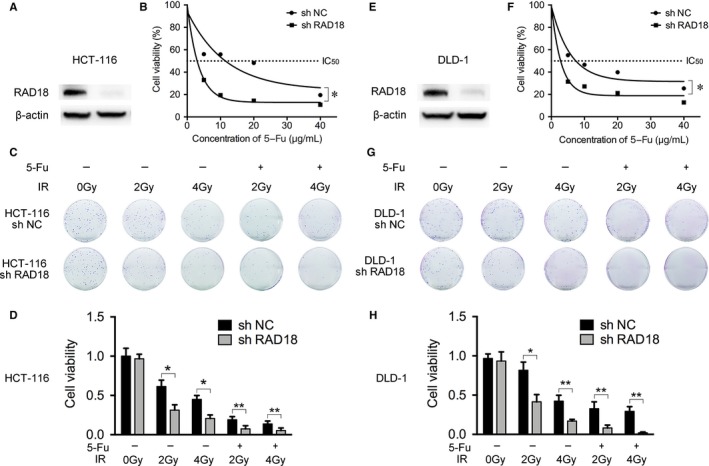
Inhibition of RAD18 increased chemoradiosensitivity of rectal cancer cells in vitro. A, The expression levels of RAD18 in shRAD18 and shNC HCT‐116 cells were identified by western blot analysis. Endogenous β‐actin served as a loading control. B, CCK‐8 assays were operated to detect cell proliferation with 5‐Fu at different concentrations (0, 5, 10, 20, and 40 μg/mL). Downregulation of RAD18 suppressed the cell proliferation in 5‐Fu dose‐dependent manner, and the IC_50_ (μg/mL) in shRAD18 HCT‐116 cells (1.74 ± 0.33) was significantly lower than shNC HCT‐116 cells (10.58 ± 2.61) (*P < 0.05). C and D, Colony formation assays were also performed by shRAD18 HCT116 and shNC HCT116 cells. The cells were treated with (0, 2, 4 Gy) ionizing radiation (IR) alone or pretreated with 5‐Fu of IC_50_ for 24 hours followed by (2, 4 Gy) IR colonies containing ≥50 cells were counted. Inhibition of RAD18 decreased clonogenic survival of rectal cancer cells in both situations of IR alone and concurrent chemoradiation. Error bars represent standard deviations (*P < 0.05, **P < 0.01). E, The expression levels of RAD18 in shRAD18 and shNC DLD‐1 cells were also identified by western blot analysis. F, Downregulation of RAD18 suppressed the cell proliferation was also shown in DLD‐1 cells. The IC_50_ (μg/mL) in shRAD18 DLD‐1 cells (1.15 ± 0.34) was significantly lower than shNC DLD‐1 cells (7.74 ± 0.72) (*P < 0.05). G and H, Colony formation assays were performed as before in DLD‐1 cells. Inhibition of RAD18 in DLD‐1 cells decreased clonogenic survival in all treatment groups (*P < 0.05, **P < 0.01). All experiments were carried out in triplicate and repeated three times

To inquire into the functional effects of RAD18 on biological behaviors of rectal cancer cell, we transfected RAD18‐targeting shRNA and control into adopted rectal cancer cells, HCT‐116, and DLD‐1 to generate specific knockdown‐RAD18 cell models. Western blotting analysis verified the RAD18 expression (Figure 3A,E). The cells were named as shRAD18 group and shNC group, respectively.

To validate the function of RAD18 in resistance to 5‐Fu, we detected the survival rate of rectal cancer cells exposed to different doses of 5‐Fu by CCK‐8 proliferation assay. The cell viability rate revealed that knockdown of RAD18 notably restrained the proliferation of HCT‐116 and DLD‐1 after exposed to 5‐Fu (Figure 3B,F). The IC_50_ (μg/mL) in shRAD18 HCT‐116 and shNC HCT‐116 cells were 1.74 ± 0.33 and 10.58 ± 2.61, respectively, and the similar results were shown in DLD‐1 cells: the IC_50_ (μg/mL) in shRAD18 DLD‐1 and shNC DLD‐1 cells were 1.15 ± 0.34 and 7.74 ± 0.72, separately. Colony formation assay was used to reveal the character of RAD18 in resistance to irradiation in rectal cancer cell lines. As expected, cell survival rate was reduced obviously in shRAD18 groups in a dose‐dependent manner. Further, we explore the relationship between RAD18 expression in rectal cancer cell lines and concurrent chemoradiation sensitivity. Cells were pretreated by 5‐Fu or PBS for 24 hours before being exposed to irradiation. As shown in Figure 3C,G,D,H, we found that 5‐Fu precisely sensitized HCT‐116 and DLD‐1 cells to irradiation. Cell colony formation in the combined‐chemoradiation group was remarkably decreased compared to irradiation‐alone group. Regardless of the irradiation dose, the numbers of cell colony formation in the shRAD18 groups were always much lower than the shNC groups. In summary, these results revealed that knockdown of RAD18 markedly improved the sensitivity of rectal cancer cells to chemotherapy, radiotherapy, and concurrent chemoradiotherapy.

### 3.3 Inhibition of RAD18 increased chemoradiation‐induced apoptosis of rectal cancer cells in vitro through activating caspase‐9‐caspase‐3‐dependent apoptotic pathway

**Figure 4:**
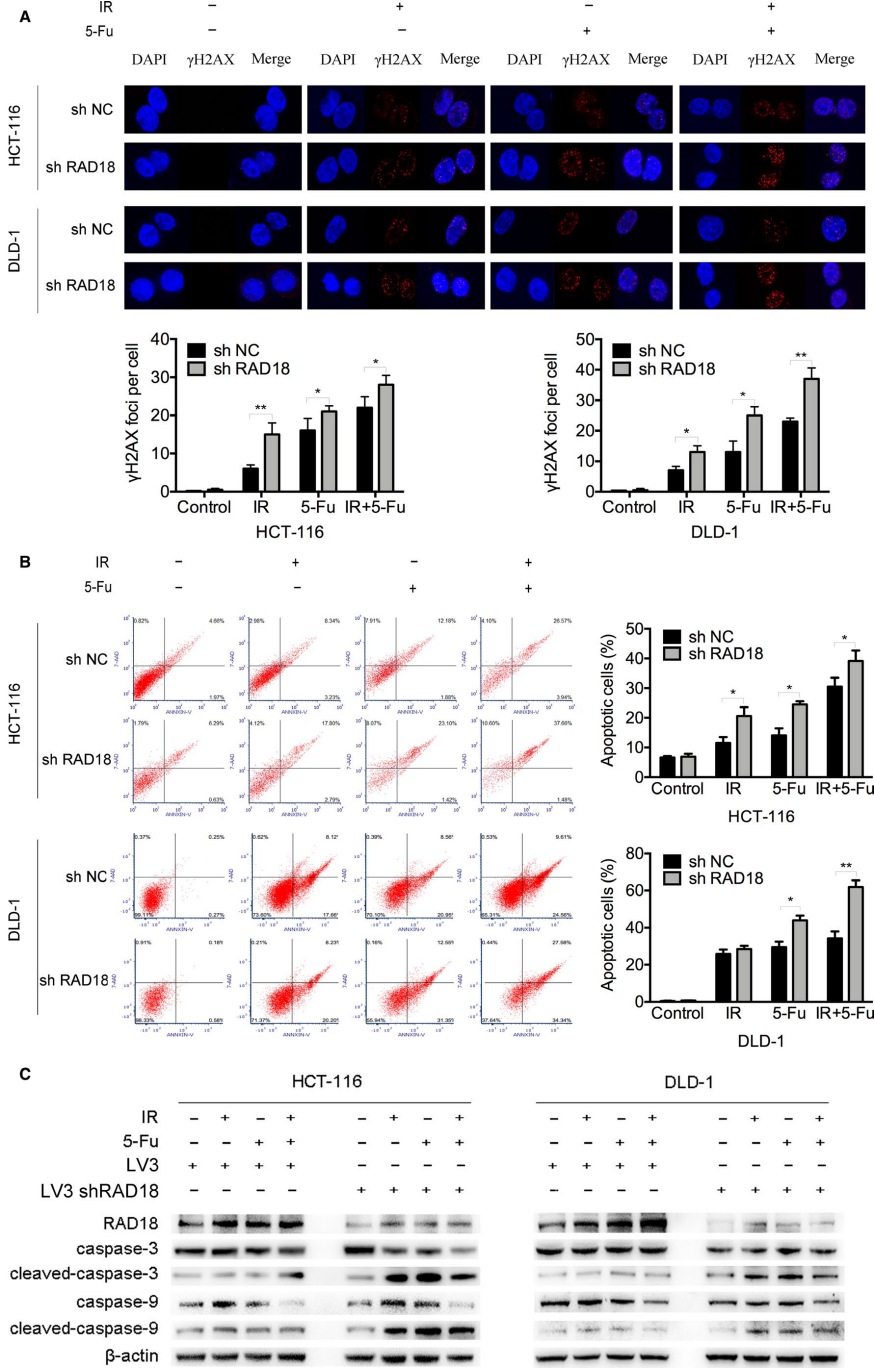
Inhibition of RAD18 increased chemoradiation‐induced apoptosis of rectal cancer cells in vitro through activating caspase‐9‐caspase‐3‐dependent apoptotic pathway. A, The assessments of γH2AX foci in shRAD18 HCT116 and shNC HCT116 cells, shRAD18 DLD‐1 and shNC DLD‐1 cells after different treatments were shown (*P < 0.05, **P < 0.01). Cells were dealt with IR (4 Gy), 5‐Fu (IC_50_), and IR (4 Gy) combined with 5‐Fu (IC_50_), respectively. A blank control group was also set up. After 4 h, the cells were fixed and incubated with anti‐γH2AX antibody. The fluorescence of γH2AX was detected. B, Cell apoptosis in shRAD18 HCT116 and shNC HCT116 cells, shRAD18 DLD‐1 and shNC DLD‐1 cells was assessed by Annexin V and 7‐AAD (*P < 0.05, **P < 0.01). Cells were treated as above and collected after 4 h and then stained with Annexin V/7‐AAD. The apoptotic cells were detected and compared. C, Western blotting was performed to detect RAD18, caspase‐9, cleaved‐caspase‐9, caspase‐3, and cleaved‐caspase‐3 expression in shRAD18 HCT116 and shNC HCT116 cells, shRAD18 DLD‐1 and shNC DLD‐1 cells. Endogenous β‐actin was used as loading control. Cells were treated as above and collected after 4 h. All experiments were repeated three times

For further study, we evaluated whether RAD18 depletion‐induced hypersensitivity to chemoradiation was attributed to more double‐strand breaks in rectal cancer cells. Cells were dealt with ionizing radiation (IR), 5‐Fu, and IR combined with 5‐Fu, respectively. A blank control group was also set up. Immunofluorescent staining of γH2AX, which is an early marker for double strand breaks, was performed. As shown in Figure 4A, numbers of γH2AX foci observed in shRAD18 cells were increased observably compared to shNC cells in all treatment groups. To determine the mechanism of which inhibited RAD18 facilitates the sensitivity of rectal cancer cell to chemoradiation, the cell apoptosis was assessed. Cells were treated as above and then stained with Annexin V/7‐AAD. As shown in Figure 4B, the cell apoptotic rates were markedly increased in shRAD18 cells compared to shNC cells of all treatment groups. Beyond that, we performed western blot assays to verify the expression level of two key apoptosis‐associated proteins including caspase‐9 and caspase‐3. As shown in Figure 4C, the levels of cleaved fragments of caspase‐9 and caspase‐3 were markedly increased in the shRAD18 cells compared to the shNC cells of all treatment groups. In contrast, the pro‐caspase‐9 and pro‐caspase‐3 in shRAD18 cells were markedly reduced than shNC cells. In addition, cells treated with a combination of 5‐Fu and irradiation were prone to have more DNA damage focus, cell apoptosis, and apoptotic proteins expression compared to 5‐Fu or IR alone. These findings showed that downregulation of RAD18 conferred more chemoradiation‐mediated apoptosis in rectal cancer cells via activation of caspase‐9‐caspase‐3‐dependent apoptotic pathway.

### 3.4 RAD18 improved the sensitivity of rectal cancer cells to chemoradiation in vivo

**Figure 5:**
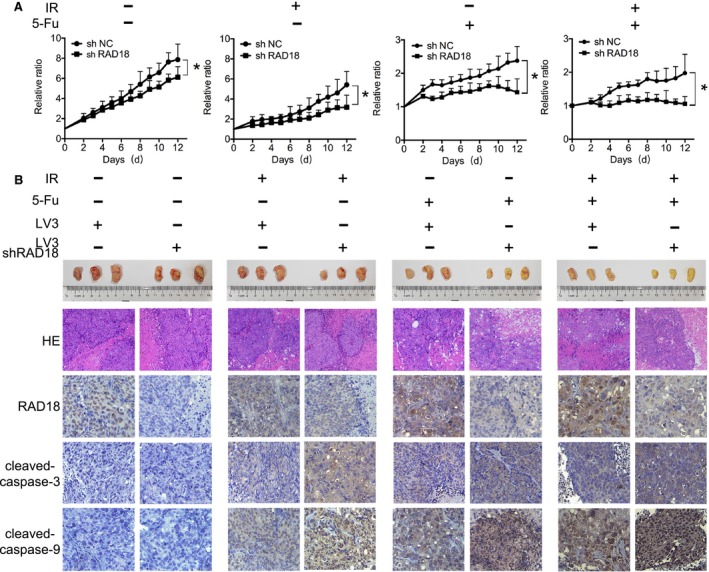
RAD18 increased the sensitivity of rectal cancer cells to chemoradiation in vivo. A, The effects of 5‐Fu or/and IR on tumor growth of xenograft nude mouse with different RAD18 expression were shown. Nude mice were firstly grouped into four groups (six mice per group): group 1: control group; group 2: IR alone (10 Gy for 1 time); group 3:5‐Fu alone (10 mg/kg intraperitoneal injection for three consecutive days); group 4: IR and 5‐Fu (10 Gy for 1 time and intraperitoneal injection of 10 mg/kg 5‐Fu for three consecutive days prior to the IR). In each group, three mice were injected with shRAD18 HCT‐116 cells and another three were injected with shNC HCT‐116 cells. The tumor sizes were measured every 2 d following IR The initial mouse tumor size was designated as 1, and subsequent tumor sizes were quantified via comparison to the initial tumor size. Tumor growth of shRAD18 HCT‐116 model has been significantly inhibited in a time‐dependent manner following treatment (*P < 0.05). B, All of the mice were executed after 2 wk. Tumors were evaluated by hematoxylin and eosin (HE) staining and immunohistochemical analysis of RAD18, cleaved‐caspase‐9 and cleaved‐caspase‐3 in tumor extracts (×200 magnification)

Based on the results in vitro, we further confirmed the regulatory role of RAD18 in chemoradiation sensitivity in vivo. The shRAD18 and shNC HCT‐116 cells were injected into female nude mice subcutaneously. After 2 weeks, mice were executed and tumor tissues were detected by HE staining. As expected, the tumor growth rate of shRAD18 xenograft model was observably restrained by chemoradiation: the mean volume (cm^3^) of tumor was much smaller in shRAD18 xenograft model than shNC xenograft model. Tumor volume was further reduced when xenograft model was treated with a combination of 5‐Fu and irradiation compared to 5‐Fu or irradiation alone (Figure 5A). Although the tumor growth curves between shRAD18 group and shNC group were also significantly different in group 1 (control group), the tumor growth curves in shRAD18 groups of different treatments showed a downward trend to depict the function of chemotherapy or radiotherapy. In addition, we performed immunohistochemistry to determine the expression level of caspase‐9 and caspase‐3 in tumor tissues. A concomitant rising tendency of cleaved‐caspase‐9 and cleaved‐caspase‐3 level was exhibited in all treatment groups when RAD18 expression was suppressed (Figure 5B), confirming the potential correlation between inhibited RAD18 and chemoradiation‐induced cell apoptosis. Taken together, these consequences implied that downregulation of RAD18 enhanced the chemoradiosensitivity of rectal cancer cells in vivo through activating the caspase‐9‐caspase‐3‐related apoptotic pathway.

## 4 DISCUSSION

The different curative effects of nCRT promoted the researchers to find predictive biomarkers to develop individual treatment patterns for better prognoses of LARC patients. Over the past decades, endoscopy, MRI, and serum carcinoembryonic antigen have been the most commonly used prediction models for tumor responses after nCRT.^8^ These models guide clinicians to select the best treatment for each LARC patient. However, several methods have shown some limitations in accuracy. Thus, more predictive molecular markers and combined models with high accuracy are needed to better predict the response of nCRT. Here, by comparing the expression levels of RAD18 in pretreatment biopsy specimens of LARC, we found that RAD18 was highly expressed in tumors that was resistance to nCRT (Figure 2) implying a potential role of RAD18 as a predictive biomarker for nCRT efficiency in LARC.

RAD18 is confessed as a DNA repair protein, initially identified in yeast where it acted with RAD6 in DNA double‐strand breaks repair.^19^, ^20^ Many previous studies have revealed that abnormal expression of RAD18 could be found in different cancer cells.^9^, ^21^ High RAD18 expression enhances translesion synthesis and double‐strand breaks repair,^22^, ^23^, ^24^ leading to tolerance of DNA damage and replication stress,^25^, ^26^ thus resulting in chemo‐resistance.^27^, ^28^ High RAD18 level in cancer cells also represents extreme resistance to IR by regulating DNA damage‐dependent checkpoint and G2/M checkpoint.^15^, ^26^ Consistent with this, we found that Rad18 was overexpressed in colorectal cancer tissue and expressed weakly in normal tissue according to TCGA samples (Figure S1). We have shown that inhibited RAD18 promoted the sensitivity to chemoradiation in vitro by CCK‐8 proliferation assay and colony formation assay. A xenograft nude mouse model confirmed that inhibition of RAD18 enhanced the sensitivity to chemoradiotherapy in vivo. Therefore, RAD18 participated in mediating the response to chemoradiotherapy in rectal cancer.

Apoptosis is a physiological process of endogenous programmed cell death, mediated by varieties of stimulus including chemoradiotherapy.^29^ 5‐Fu and irradiation may exert their anticancer effects through activating cell apoptosis signaling.^30^, ^31^, ^32^ The prevention of apoptosis has been reported to be an important cause in chemoradiation resistance.^33^, ^34^ We have shown that inhibited RAD18 promoted the sensitivity of 5‐Fu and irradiation in rectal cancers. We further explored whether inhibited RAD18 could facilitate cell apoptosis, thus promoting the response to 5‐Fu and irradiation. Both control and experimental cells exposed to 5‐Fu or/and irradiation were assessed by immunofluorescence assay and apoptosis assay. As predicted, depletion of RAD18 in rectal cancer cell lines led to the accumulation of chemoradiation‐induced genomic damage and promoted cell apoptosis significantly, whereas the DNA damage sites and cell apoptosis in control group were relatively few (Figure 4A,B). The process of cell apoptosis is primarily activated by caspase family under the stimulation of death.^34^, ^35^ Caspase‐9 and caspase‐3 are vital to the apoptotic pathway as they are, respectively, the key initiator and the executor in charge of definite cleavage of cellular components.^36^, ^37^ Then western blot confirmed that the cleaved‐caspase‐9 and cleaved‐caspase‐3 expression levels in inhibited RAD18 cells were much higher than cells in control groups after exposed to 5‐Fu and irradiation (Figure 4C). The same conclusions have been verified in vivo (Figure 5B). It seemed that inhibition of RAD18 level functioned as an accelerator promoting the activation of caspase‐9‐caspase‐3‐dependent apoptotic signaling responding to chemoradiation, thus effectively facilitated the apoptosis of rectal cancer cells. In other words, inhibited RAD18 may increase sensitivity to nCRT in LARC patients through activating caspase‐9‐caspase‐3‐dependent cell apoptosis process.

In conclusion, our study revealed that RAD18 may function as a potential predictive marker for nCRT sensitivity of LARC patients and may be of latent value in therapeutic target against LARC, thus improving the prognosis of LARC patients.

## CONFLICT OF INTEREST

The authors declare no conflict of interest.
